# An Exploratory Investigation of Endotoxin Levels in Novice Long Distance Triathletes, and the Effects of a Multi-Strain Probiotic/Prebiotic, Antioxidant Intervention

**DOI:** 10.3390/nu8110733

**Published:** 2016-11-17

**Authors:** Justin D. Roberts, Craig A. Suckling, Georgia Y. Peedle, Joseph A. Murphy, Tony G. Dawkins, Michael G. Roberts

**Affiliations:** 1 Department of Life Sciences, Faculty of Science and Technology, Anglia Ruskin University, Cambridge Campus, Cambridge CB1 1PT, UK; craig.suckling@pgr.anglia.ac.uk (C.A.S.); georgia.peedle@student.anglia.ac.uk (G.Y.P.); 2 College Lane, School of Life and Medical Sciences, University of Hertfordshire, Hatfield, Hertfordshire AL10 9AB, UK; jamurphy123@gmail.com (J.A.M.); m.g.roberts@herts.ac.uk (M.G.R.); 3 Cardiff School of Sport, Cardiff Metropolitan University, Cyncoed Campus, Cyncoed Road, Cardiff CF23 6XD, UK; tdawkins@cardiffmet.ac.uk

**Keywords:** endotoxemia, probiotics, prebiotics, antioxidants, triathlon

## Abstract

Gastrointestinal (GI) ischemia during exercise is associated with luminal permeability and increased systemic lipopolysaccharides (LPS). This study aimed to assess the impact of a multistrain pro/prebiotic/antioxidant intervention on endotoxin unit levels and GI permeability in recreational athletes. Thirty healthy participants (25 males, 5 females) were randomly assigned either a multistrain pro/prebiotic/antioxidant (LAB^4^_ANTI_; 30 billion CFU·day^−1^ containing 10 billion CFU·day^−1^ *Lactobacillus acidophilus* CUL-60 (NCIMB 30157), 10 billion CFU·day^−1^ *Lactobacillus acidophillus* CUL-21 (NCIMB 30156), 9.5 billion CFU·day^−1^ *Bifidobacterium bifidum* CUL-20 (NCIMB 30172) and 0.5 billion CFU·day^−1^ *Bifidobacterium animalis* subspecies *lactis* CUL-34 (NCIMB 30153)/55.8 mg·day^−1^ fructooligosaccharides/ 400 mg·day^−1^ α-lipoic acid, 600 mg·day^−1^ *N*-acetyl-carnitine); matched pro/prebiotic (LAB^4^) or placebo (PL) for 12 weeks preceding a long-distance triathlon. Plasma endotoxin units (via *Limulus* amebocyte lysate chromogenic quantification) and GI permeability (via 5 h urinary lactulose (L): mannitol (M) recovery) were assessed at baseline, pre-race and six days post-race. Endotoxin unit levels were not significantly different between groups at baseline (LAB^4^_ANTI_: 8.20 ± 1.60 pg·mL^−1^; LAB^4^: 8.92 ± 1.20 pg·mL^−1^; PL: 9.72 ± 2.42 pg·mL^−1^). The use of a 12-week LAB^4^_ANTI_ intervention significantly reduced endotoxin units both pre-race (4.37 ± 0.51 pg·mL^−1^) and six days post-race (5.18 ± 0.57 pg·mL^−1^; *p* = 0.03, ηp^2^ = 0.35), but only six days post-race with LAB^4^ (5.01 ± 0.28 pg·mL^−1^; *p* = 0.01, ηp^2^ = 0.43). In contrast, endotoxin units remained unchanged with PL. L:M significantly increased from 0.01 ± 0.01 at baseline to 0.06 ± 0.01 with PL only (*p* = 0.004, ηp^2^ = 0.51). Mean race times (h:min:s) were not statistically different between groups despite faster times with both pro/prebiotoic groups (LAB^4^_ANTI_: 13:17:07 ± 0:34:48; LAB^4^: 12:47:13 ± 0:25:06; PL: 14:12:51 ± 0:29:54; *p* > 0.05). Combined multistrain pro/prebiotic use may reduce endotoxin unit levels, with LAB^4^_ANTI_ potentially conferring an additive effect via combined GI modulation and antioxidant protection.

## 1. Introduction

Participation trends, including “recreational athletes”, in multi-sport and ultra-endurance events have increased in recent years [1,2]. Symptoms associated with gastrointestinal (GI) distress (e.g., cramping, diarrhoea, nausea, and abdominal pain) are estimated to occur in 25%–90% of endurance athletes, and are often cited as reasons for non-completion [3,4,5]. In preparation for such events, exercise-related GI symptoms may go unreported, which could impact on training efficiency and race completion. It has been shown that exercise induced GI hypoperfusion may provoke transient damage to the gut epithelium [6], with one study demonstrating that 30 min of running at 80% of peak oxygen uptake (VO_2_peak) significantly increased luminal permeability in healthy volunteers [7].

Mechanistically, prolonged or strenuous exercise may increase key phosphorylation enzymes [8], disrupting the tight junction proteins claudin (influenced by protein kinase A) and occludin (influenced by both protein kinase C and tyrosine kinase). Acute changes in tight junction permeability and paracellular transport may lead to a greater prevalence of systemic lipopolysaccharides (LPS). LPS from gram-negative intestinal bacteria may provoke immune responses and endotoxin-associated symptoms characteristic of GI complaints often experienced in runners [8]. Despite this, research is relatively sparse on whether prolonged training or ultra-endurance events actually result in elevated LPS, particularly in more “recreationally active” athletes; or whether targeted nutrition strategies offer beneficial support.

In one study, 68% of highly trained athletes taking part in a long-distance triathlon reported with endotoxin levels of 5–15 pg·mL^−1^ in the first 16 h post-event, corresponding with elevated cytokine responses in the same period [9]. In contrast, 81% of runners requiring medical attention at the end of an ultra-marathon were found to have LPS concentrations >100 pg·mL^−1^ [10], with 80.6% of these athletes reporting GI symptoms (nausea, diarrhoea, and vomiting). LPS concentrations at or above these levels have been more commonly associated in patients with Crohn’s disease [11] and sepsis [12].

The term “mild endotoxemia” has been used to depict an acute elevation in LPS from endurance exercise by several authors [9,13,14], but may well reflect normal or transient levels of circulatory LPS. It has also been shown that LPS responses to exertional heat stress may be significantly higher in less trained individuals [13], but still within normal limits. Conversely, one study reported an average increase in resting LPS levels of 60 pg·mL^−1^ across a five-stage ultra-run, with daily (pre–post stage) average LPS changes of 30 pg·mL^−1^ [15]. Despite such diversity, the potential for exercise related endotoxin-mediated cytokinemia may explain individual susceptibility to GI symptoms and recovery from endurance exercise. If prevalent, the presence of, and repeated exposure to, “low grade” LPS (ranging from ~10 to 50 pg·mL^−1^ or higher [16]) may promote a mild inflammatory state which could be detrimental to the longer term health of recreational athletes who regularly engage in exercise.

Probiotic bacteria, particularly the gram-positive genera *Lactobacillus* and *Bifidobacterium* species, are known to modify GI microbiota [17,18,19], and have been shown to reduce GI episode severity [20] and respiratory tract infections commonly associated with training [21]. However, therapeutic benefits of probiotics are highly strain specific. As example, the use of *Lactobacillus casei* strain Shirota in one study, significantly increased natural killer cell cytolytic activity in healthy volunteers [22], whereas combined *Streptococcus thermophilus* FP4/*Bifidobacterium breve* BR03 was recently shown to reduce circulating IL-6 in response to muscle damaging exercise [23] elsewhere. In clinical trials, a multistrain high dose probiotic (LAB^4^—containing *Lactobacillus acidophilus* CUL60 and CUL21, *Bifidobacterium lactis* CUL34 and *Bifidobacterium bifidum* CUL20), resulted in significant improvements in irritable bowel syndrome responses [24] and prevented an increase in antibiotic resistant enterococci [25]. Chronic multistrain interventions have also been shown to reduce faecal zonulin levels by ~25% in endurance trained athletes, demonstrating improved GI barrier integrity [26]. The inclusion of *Bifidobacterium species* and prebiotics (e.g., fructo-oligosaccharides, inulin, pectin) in such formulas may also play an important role in short-chain fatty acid production, which may also support epithelial integrity [27].

Antioxidants nutrients such as α-lipoic acid, *N*-acetyl-carnitine, vitamin C, quercetin, resveratrol, and curcumin may also provide important roles in minimizing epithelial disruption [28,29,30,31], associated with elevated oxidative stress from GI hypoperfusion. Alpha lipoic acid in particular is proposed to act as a multi-functional antioxidant, regenerating endogenous glutathione, and minimising GI mucosal injury [31,32,33]. The aims of this exploratory study were therefore: (i) to assess endotoxin levels and GI permeability in recreational athletes training for and taking part in their first long distance triathlon; and (ii) to assess the potential benefits of a 12-week multistrain pro/prebiotic/antioxidant strategy on GI symptoms, endotoxin levels and race time compared to a control group.

## 2. Materials and Methods

### 2.1. Participants

Following study approval from the University of Hertfordshire Life and Medical Sciences Ethics Committee (LMS/SF/UH/00011), and power calculation assessment for sample size (G*power3, Dusseldorf [34]; using α = 0.05; 1 − β = 0.80; based on observed data [9,14]), thirty recreationally active participants (25 males, 5 females; M ± SE: age 35 ± 1 years; weight: 76.52 ± 2.20 kg; initial VO_2_max: 48.93 ± 0.99 mL·kg^−1^·min^−1^) were randomly invited to take part in an intervention study which took place in the final 12 weeks of a nine month progressive training programme. All participants provided written, informed consent, and satisfactorily completed a general health screen prior to study inclusion. Participant characteristics are displayed in Table 1, with no observed differences between intervention groups for age, height, weight, bodyfat or VO_2_max.

*Pre-screening:* At the start of the nine month training programme, all participants underwent full screening including suitability assessment from their General Practitioner, a 12-lead electrocardiogram to assess for potential underlying cardiac abnormalities, and completion of a standard incremental maximal stress test (using a Computrainer erogometer system, RaceMate Inc., Seattle, WC, USA) for the assessment of maximal oxygen consumption (using a Metalyser 3B automated gas-analyser; Cortex Biophysik, Leipzig, Germany). In addition, routine assessment of height (Seca 200 stadiometer, Hamburg, Germany), body mass (Seca 780, Hamburg, Germany) and body composition (Tanita Body Segmental Analyser 418-BC, Tokyo, Japan) was undertaken. Participants were required to have no previous experience of long distance triathlons, be recreationally active (defined as general exercise activity 1–3 times per week) and have basic proficiency in swimming, cycling and running disciplines. As a means to further quantify “recreationally active”, participants were required to have a relative maximum oxygen uptake of 30–50 mL·kg^−1^·min^−1^ for women, and 35–55 mL·kg^−1^·min^−1^ for men during pre-screening testing. Participants were excluded if there was any history (including familial) of cardiovascular abnormalities (including coronary heart disease) and diabetes; or any known blood related disorders.

### 2.2. Experimental Design and Procedures

In a randomized, repeated-measures, double-blind, placebo controlled study design, participants attended the Human Physiology Laboratory, University of Hertfordshire 12 weeks prior to undertaking a long distance triathlon (Barcelona Challenge Triathlon) comprising a 3.8 km sea swim, 180.0 km road cycle course and a 42.2 km marathon run. Although participants had no prior experience to this triathlon distance, they had all adhered to a standardized training programme for the previous six months as part of a larger training cohort. General training progression (“recreationally trained”) from the previous six months was assessed prior to the intervention study using the same incremental test procedure and equipment (including anthropometrical measures) as for pre-screening (see Table 1). Thereafter, participants attended the laboratory on three occasions: baseline (Week 0), pre-race (Week 12) and post-race (six days post) for blood and urine sampling as described below. Due to constraints with field based sampling, and varying participant travel arrangements, the post-race timepoint (six days) was selected for consistency and to assess whether any previous patterns were still evident during the longer term recovery period.

*Blood sampling:* Participants were requested to rest the day before all test sessions. Upon arrival, a fasted, venous wholeblood sample was collected from participants by a qualified phlebotomist into duplicate 4 mL K_3_EDTA vacutainers (Greiner Bio-One GmbH, Kremsmunster, Austria). Samples were centrifuged for 10 min at 3000 rpm, with aliquotted plasma pipetted into sterile, nonpyrogenic, polypropylene cyrovials (Fisherbrand, Fisher Scientific, Loughborough, UK) and immediately frozen at −80 °C for later assessment of resting endotoxin units and IgG endotoxin-core antibodies.

*Urine sampling:* Assessment of GI permeability was assessed via 5 h recovery of urinary lactulose and mannitol via a standard sugar absorption test [35]. Briefly, upon arrival, participants provided a urine sample with total volume assessed, and then (following blood sampling) consumed a standardized 100 mL test drink containing 5 g lactulose solution (Sandoz Ltd., Camberley, Surrey, UK), 2 g mannitol (Mannitol powder: 99.86% pure certified, Blackburn Distributions Ltd., Nelson, Lancashire, UK) and 40 g of sucrose (Tate and Lyle, London, UK). For the first two hours post consumption, participants were not allowed to eat or drink, and thereafter could eat/ drink as normal (with the exception of refined/sugary products or drinks). Over a five hour period, participants collected total urine output into 3 L polyethylene opaque beakers (Sarstedt, Numbrecht, Germany). With total sample volume assessed, duplicate urine samples were aliquotted into sterile cryovials and immediately frozen at −80 °C for later assessment of saccharide recovery.

### 2.3. Biochemical Assays

*Endotoxin unit assessment:* Quantification of endotoxin units was derived from plasma samples using an established endpoint chromogenic assay method (Pierce^®^ LAL Chromogenic Endotoxin Quantitation Kit, Thermo Fisher Scientific, Waltham, MA, US). After thawing to room temperature and sample preparation, 50 µL aliquots were added to an endotoxin-free microtitre plate and incubated at 37 °C for 5 min. Following this, 50 µL aliquots of *Limulus* amoebocyte lysate (LAL) were added to each well, the plate gently shaken for 10 s, and re-incubated at 37 °C for 10 min. At exactly 10 min, 100 µL aliquots of chromogenic substrate solution was added to each well, the plate gently shaken for 10 s, and then further re-incubated at 37 °C for 6 min. At this point, 50 µL aliquots of stop reagent (25% acetic acid) was added to each well, and the plate gently shaken for 10 s. Samples were read on a spectrophotometer at an absorbance of 405 nm (Victor 3 multilabel plate reader, PerkinElmer Inc., Llantrisant, UK) and referenced against a calibration curve based on dilutions of an *Escherichia coli* (*E. coli*) endotoxin standard (011:B4; vial concentration 26 EU·mL^−1^) with non-incubated mock reaction controls taken into consideration. Values of quantified endotoxin units (EU·mL^−1^) were then converted to pg·mL^−1^.

*IgG Endotoxin-core Antibody Assessment:* IgG endotoxin-core antibodies (IgG anti-EU) were measured from plasma samples via solid-phase ELISA (EndoCab^®^ IgG, Hycult Biotech, Uden, The Netherlands). Reagents were prepared in accordance with the manufacturer’s instructions at room temperature. Plasma samples were thawed to room temperature and diluted 200-fold using the supplied dilution buffer. Following this, 100 μL aliquots of the standard or prepared sample were carefully pipetted into microtitre wells coated with endotoxin rough-lipopolysaccharides, the microtitre plate then covered and incubated at 37 °C for 60 min. The plate was then washed four times manually, with 200 μL of supplied washer buffer added to each microtitre well during each wash cycle. Following this, 100 μL of diluted conjugate (streptavidin-peroxidase) was added to each well to bind the captured endotoxin core-antibodies. The plate was then covered and incubated at 37 °C for 60 min, before being manually washed a further four times with washer buffer. Then, 100 μL aliquots of tetramethylbenzidine (TMB) were added to each microtitre well, the plate covered and incubated at room temperature for 30 min avoiding exposure to sunlight. The reaction was then stopped by addition of 100 μL aliquots of oxalic acid to each well. Samples were read on a spectrophotometer at an absorbance of 450 nm (Victor 3 multilabel plate reader, PerkinElmer Inc., Llantrisant, UK) and referenced against a calibration curve (logarithmic scale) based on dilutions of a reconstituted human EndoCab IgG standard. Values are presented in standard median units (MU·mL^−1^).

*GI Permeability Assessment:* Following sample thawing and preparation, assessment of saccharide recovery was performed via enzymatic method assays for lactulose and mannitol using a Randox RX Monza semi-automated, flow cell based clinical chemistry analyser (Randox Ltd., Country Antrim, UK). Briefly, for lactulose, reagents were prepared in accordance with the manufacturer’s instructions at room temperature (INstruchemie BV, Delfzil, Netherlands). Sample preparation involved 50 μL urine aliquots being mixed with 50 μL dissociation buffer and 5 μL galactosidase reagent, incubated at 37 °C overnight, and centrifuged at 2000 rpm for 5 min. Additionally, a non-incubated control was also prepared to account for NADPH already present in the sample. Thereafter, 200 μL of lactulose buffer reagent was carefully pipetted into centrifuge tubes and mixed with 5 μL sample, incubated at 37 °C for 5 min, and read at an absorbance of 340 nm (reading A1). Following this, 50 μL start reagent was added to the sample, mixed and incubated at 37 °C for 10 min and read at an absorbance of 340 nm (reading A2). Recovered lactulose (mmol·L^−1^) was calculated taking into consideration pre-incubated and non-incubated samples against standard, blank and quality control samples.

For mannitol assessment, reagents were prepared in accordance with the manufacturer’s instructions at room temperature (INstruchemie BV, Delfzil, The Netherlands); and a reference curve generated from dilutions of a 20 mmol·L^−1^ mannitol standard. Samples were prepared by mixing 3 μL urine aliquots with 200 μL NAD buffer reagent and incubating at 37 °C for 5 min. From this 60 μL diluted start reagent was added to the sample, mixed and incubated for a further 37 °C for 10 min. Samples were read at an absorbance of 340 nm, and recovered mannitol (mmol·L^−1^) calculated against a standard reference taking into consideration blank and quality control samples.

### 2.4. Nutritional Interventions and Diaries

*Nutritional interventions:* Following baseline assessment, participants were allocated, in a double-blinded manner, to one of three intervention groups using a random number generator approach. Participants were provided with a 90-day supply of either: capsulated (hydroxypropyl methylcellulose) multistrain probiotic/prebiotic/antioxidant (LAB^4^_ANTI_), matched pro/prebiotic (LAB^4^) or placebo (PL) in opaque, sealed pots with instructions for daily ingestion timing. This allocation covered the 12-week pre-race period and the six-day post-race period. Intervention supplementation was provided by Biocare Ltd. (Birmingham, UK) for commercial use, with products pre-capsulated by the manufacturer. Placebo supplements were prepared within our laboratory using the same size hydroxypropyl methylcellulose capsules.

For both LAB^4^ and LAB^4^_ANTI_, participants were instructed to consume one multistrain pro/prebiotic capsule daily in the evening with food. Each multistrain capsule contained 150 mg·day^−1^ *Lactobacillus acidophilus* (10 billion CFU·day^−1^, *Lactobacillus acidophilus* CUL-60 [NCIMB 30157] and 10 billion CFU·day^−1^ *Lactobacillus acidophillus* CUL-21 [NCIMB 30156]), 16.8 mg·day^−1^ *Bifidobacterium bifidum* and *lactis* (9.5 billion CFU·day^−1^, *Bifidobacterium bifidum* CUL-20 [NCIMB 30172] and 0.5 billion CFU·day^−1^ *Bifidobacterium animalis subspecies lactis* CUL-34 [NCIMB 30153]), and 55.8 mg·day^−1^ fructooligosaccharides (Bio-Acidophilus Forte, Biocare Ltd., Birmingham, UK). For those assigned to PL, participants were instructed to consume one placebo capsule daily in the evening with food, containing 200 mg cornflour.

For LAB^4^_ANTI_, participants additionally consumed two capsules in the morning with breakfast (each capsule contained 200 mg α-lipoic-acid and 300 mg of *N*-acetyl-carnitine hydrochloride; Acetyl Carnitine and Alpha Lipoic Acid formulation, Biocare Ltd., Birmingham, UK). For control consistency between groups, those assigned to LAB^4^ and PL were instructed to additionally consume matched cornflour placebo capsules with breakfast. Throughout the study, participants were required to not be consuming any other nutritional supplements other than glucose drinks/gels as part of endurance training. Adherence was checked via nutrition diaries and monthly briefings with all participants.

*Nutrition diaries*: Participants were requested to maintain habitual dietary intake throughout the intervention and record via weekly food diaries at the beginning and end of each month. Participants were provided with example diaries and individually instructed in diary completion, with emphasis on meal breakdown, portion size and weight, fluid intake and consumption of prescribed supplementation. Dietary analyses were undertaken using Dietplan 6.50 (Forestfield Software Ltd., West Sussex, UK) based on a seven-day representation from each month.

### 2.5. Training Monitoring, GI Questionnaires and Assessment of Race Times

*Training programme:* Over the course of the 12-week intervention, participants continued with a triathlon training programme, prescribed by an accredited Sport and Exercise Physiologist, focusing on swimming, cycling and running disciplines, as well as functional training. Training was designed to be flexible around daily activities with a requirement to achieve a minimum of 80% of the total training volume set. Training was monitored via weekly training diaries in which participants recorded exercise duration and overall session rating of perceived exertion (sRPE). Training load, training monotony and training strain were determined from a modified training method previously described (duration × sRPE [36,37]).

*GI response questionnaire:* Participants completed an overall monthly training GI response questionnaire, adapted from symptoms previously reported [4,9,38]. Participants were asked to subjectively rate their responses across four subsections (general training, endurance training (>3 h), acute (<24 h) and longer term (<72 h) recovery periods). For the training subsections the following symptoms were evaluated: urge to urinate, urge to defecate, bloating, belching, flatulence, nausea, stomach/intestinal pain or discomfort, stomach/intestinal cramping, headaches, and dizziness. For the recovery subsections, the following symptoms were evaluated: constipation and/or diarrhoea, stomach/intestinal pain or discomfort, bloating, flatulence, nausea, stomach/intestinal cramping, headaches, dizziness, mental fatigue, excessive and sweating. Collectively, this resulted in a maximum symptom count of 40. Symptoms were graded for severity according to a category scale (0 = none; 1 = low severity; 2 = moderate severity; 3 = high severity). From this, mean symptom count and symptom severity scores were assessed to evaluate the subjective impact of each intervention.

*Race times:* All participants, as entrants of the Barcelona Challenge Triathlon, were required to wear official timing chips throughout the race. Overall race times, including triathlon specific stage times (swim, bike, and run) were provided by the race director following confirmation of official final times.

### 2.6. Statistical Analyses

Statistical analyses were performed using SPSS (v22, IBM, Armonk, NY, USA). Following assessment of normality via a Shapiro–Wilk test, a 3 × 3 factorial design analysis of variance (Anova) was employed to assess treatment and time interactions, using least significant difference (LSD) post hoc evaluation. Where pertinent, within group assessment was undertaken using a general linear repeated measures Anova, with LSD post hoc evaluation. For assessment of race times between groups only, a between-group Anova was performed, with LSD post hoc analysis. GI questionnaires were assessed via chi-squared analysis. An alpha level of ≤0.05 was employed for statistical significance. Data are reported as means ± SE.

## 3. Results

### 3.1. Nutrition and Training Data

Dietary analysis comparisons for each month are shown in Table 2. No significant differences were reported between or within groups across the 12-week intervention period for energy, carbohydrate, fat or protein intake (*p* > 0.05). On average across the 12-week intervention, daily energy intake for LAB^4^_ANTI_ was 35.10 ± 1.31 kcal·kg^−1^·day^−1^ compared with 33.97 ± 1.73 kcal·kg^−1^·day^−1^ for LAB^4^ and 35.53 ± 1.66 kcal·kg^−1^·day^−1^ for PL. Macronutrient intake was also comparable, with an average fat intake of 1.45 ± 0.08 g·kg^−1^·day^−1^ for LAB^4^_ANTI_ compared 1.27 ± 0.08 g·kg^−1^·day^−1^ for LAB^4^ and 1.30 ± 0.07 g·kg^−1^·day^−1^ for PL. Likewise, average carbohydrate intake was 4.06 ± 0.22 g·kg^−1^·day^−1^ for LAB^4^_ANTI_ compared with 4.02 ± 0.24 g·kg^−1^·day^−1^ for LAB^4^ and 4.37 ± 0.31 g·kg^−1^·day^−1^ for PL. Similarly, average protein intake was comparable between pro/prebiotic groups at 1.55 ± 0.07 g·kg^−1^·day^−1^ for LAB^4^_ANTI_, 1.50 ± 0.11 g·kg^−1^·day^−1^ for LAB^4^, and non-significantly higher for PL at 1.72 ± 0.10 g·kg^−1^·day^−1^.

Training load comparisons are shown in Table 3. No significant differences were reported between groups across the intervention for training load, monotony or strain (*p* > 0.05). There was, however, a significant time interaction effect for training load (F = 16.30, *p* < 0.0001, ηp^2^ = 0.38) and training strain (F = 4.88, *p* = 0.011, ηp^2^ = 0.16). The training programme was designed to progressively build over the 12 weeks, with a peak training load in Month 2, and an increased training strain in Month 3 leading to a final taper period prior to the race. The target range (particular for training load) represents the generic range set for all participants i.e., to meet a minimum of 80% training load.

Across the intervention, all groups satisfactorily met the minimum training load. Average training loads at Month 2 were all significantly higher than Months 1 and 3, as expected (*p* < 0.0001). Interestingly, however, peak training loads at Month 2 were all greater than the high end target set at 3278 AU (arbitrary units). For LAB^4^ in particular, training load was noted at 4311 ± 348 AU (F = 8.21, *p* = 0.006, ηp^2^ = 0.58 compared to Month 1), further reflecting the increased strain (4065 ± 381 AU) in this month (F = 6.79, *p* = 0.011, ηp^2^ = 0.53). Training strain in Month 3 was noted as being lower in all groups (*p* = 0.003) compared to Month 2 and in direct comparison to the target range.

### 3.2. Endotoxin Unit (EU) Assessment

**Figure 1:**
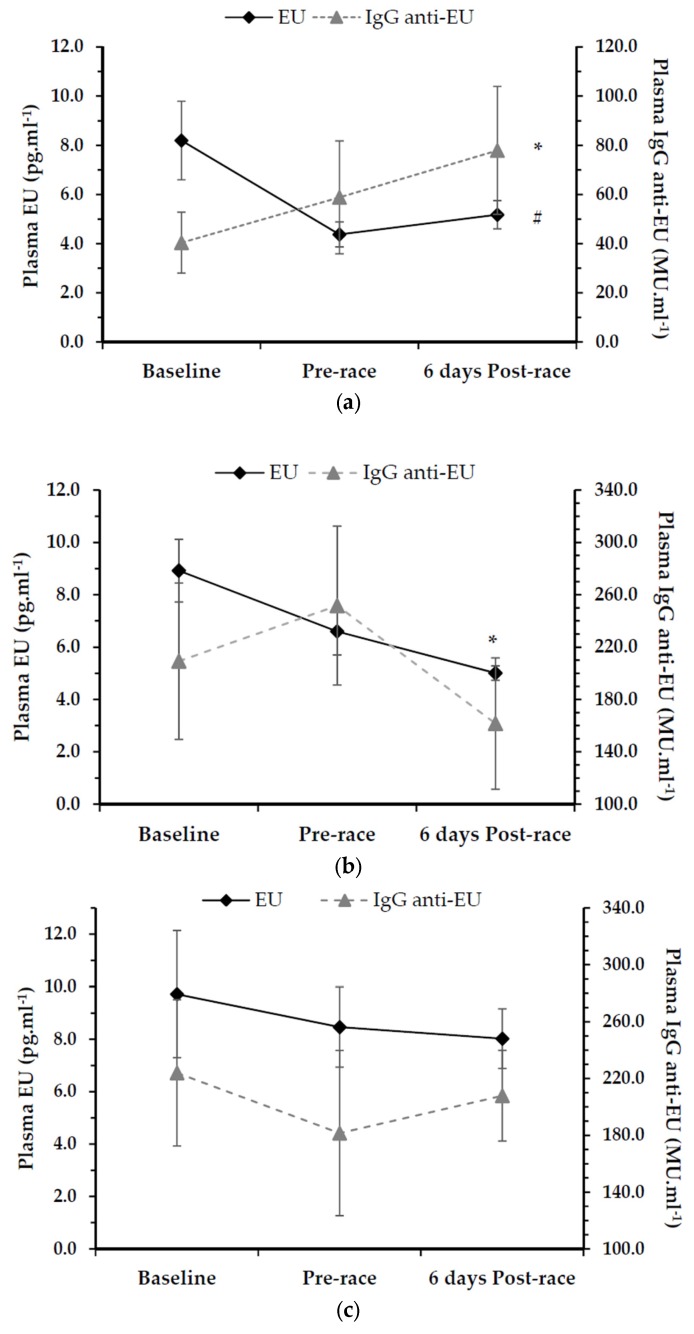
(**a**) Plasma endotoxin unit (EU) concentrations (pg·mL^−1^) and IgG endotoxin antibodies (anti-EU; MU·mL^−1^) for LAB^4^_ANTI_ group (Mean ± SE). * denotes lower IgG anti-EU values overall than both LAB^4^ and PL conditions (*p* < 0.001); # denotes significant reduction in endotoxin units over time within group (*p* = 0.03); (**b**) Plasma endotoxin unit (EU) concentrations (pg·mL^−1^) and IgG endotoxin antibodies (anti-EU; MU·mL^−1^) for LAB^4^ group (Mean ± SE). * denotes significant difference from baseline for endotoxin units within group (*p* = 0.047); (**c**) Plasma endotoxin unit (EU) concentrations (pg·mL^−1^) and IgG endotoxin antibodies (anti-LPS; MU·mL^−1^) for PL group (Mean ± SE). No significant differences reported (*p* > 0.05).

Data for endotoxin units (EU) and IgG endotoxin-core antibody assessment are shown in Figure 1a–c for LAB^4^_ANTI_, LAB^4^ and PL respectively. A significant interaction effect was reported for endotoxin units over time (F = 4.21, *p* = 0.019, ηp^2^ = 0.11) and group (F = 3.50, *p* = 0.036, ηp^2^ = 0.09) only. At baseline, whilst EU levels were highest with PL (9.72 ± 2.42 pg·mL^−1^), no significance was found in comparison to either LAB^4^_ANTI_ (8.20 ± 1.60 pg·mL^−1^) or LAB^4^ (8.92 ± 1.20 pg·mL^−1^, *p* > 0.05). EU concentrations ranged from 3.03 to 27.75 pg·mL^−1^. Within group, LAB^4^_ANTI_ resulted in a significant reduction in endotoxin units both pre-race (4.37 ± 0.51 pg·mL^−1^) and six days post-race (5.18 ± 0.57 pg·mL^−1^; F = 4.27, *p* = 0.033, ηp^2^ = 0.35). For LAB^4^, there was a significant reduction in endotoxin units over time (F = 6.04, *p* = 0.011, ηp^2^ = 0.43), with post-hoc analysis indicating EU levels of 5.01 ± 0.28 pg·mL^−1^ six days post-race being significantly lower than baseline (*p* = 0.047) only. Endotoxin unit levels for PL did not significantly differ across the intervention period or six days post-race (*p* > 0.05).

### 3.3. IgG Endotoxin-Core Antibody (Anti-EU) Assessment

Overall, a significant group interaction effect was reported for IgG anti-EU, with LAB^4^_ANTI_ demonstrating lower concentrations of IgG endotoxin core-antibodies in comparison to both LAB^4^ and PL (F = 10.82, *p* < 0.0001, ηp^2^ = 0.25) at baseline. Whilst IgG anti-EU levels remained significantly lower pre-race with LAB^4^_ANTI_ compared to LAB^4^ (*p* = 0.003), there was no statistical difference in comparison to PL. By post-race, no significant differences were reported between groups (*p* > 0.05). Within group, IgG anti-EU levels for LAB^4^_ANTI_ increased from 40.42 ± 12.39 MU·mL^−1^ at baseline, to 58.83 ± 22.94 MU·mL^−1^ pre-race in contrast to the decrease in endotoxin unit levels observed. However, the overall increase in IgG anti-EU to 77.93 ± 26.03 MU·mL^−1^ post-race was not deemed significant (F = 1.01, *p* = 0.387, ηp^2^ = 0.11) overall.

For LAB^4^, IgG anti-EU also increased from 209.23 ± 59.73 MU·mL^−1^ at baseline to 251.73 ± 60.72 MU·mL^−1^ pre-race in relative contrast to the decrease in endotoxin unit levels observed for this group. Post-race IgG anti-EU concentrations decreased to 161.61 ± 50.16 MU·mL^−1^, but overall changes were not deemed statistically significant (F = 1.95, *p* = 0.174, ηp^2^ = 0.20) for LAB^4^. In a converse manner, average plasma IgG anti-EU concentrations decreased from 223.98 ± 51.46 MU·mL^−1^ at baseline to 181.56 ± 58.19 MU·mL^−1^ pre-race with PL, returning to 207.94 ± 31.96 MU·mL^−1^ six days post-race; however, overall changes in IgG anti-EU for PL were not statistically significant (F = 0.30, *p* = 0.746, ηp^2^ = 0.04).

### 3.4. Intestinal Permeability

**Figure 2:**
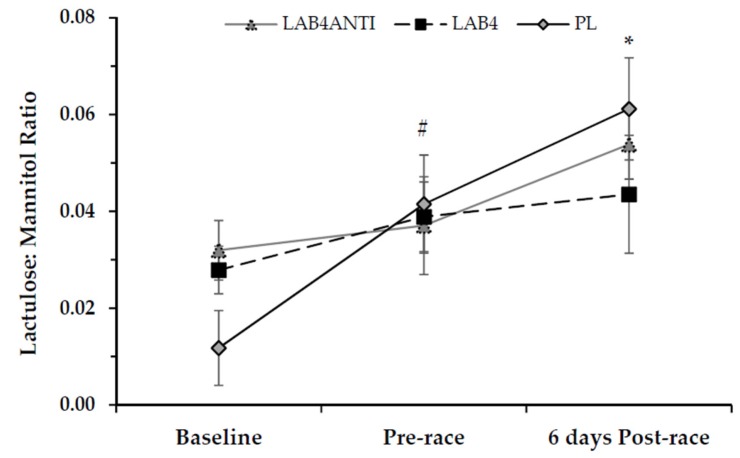
Assessment of intestinal permeability via urinary lactulose:mannitol ratio (Mean ± SE). Values measured in mmol·L^−1^. # denotes significant increase from baseline for PL only (*p* = 0.05); * denotes significant increase from baseline for PL only (*p* = 0.002).

Assessment of intestinal permeability from urinary lactulose:mannitol (L:M) ratio measurement is shown in Figure 2. GI permeability generally increased in all groups from baseline to six days post-race (F = 9.66, *p* < 0.0001, ηp^2^ = 0.21). No significant differences were reported between groups (*p* > 0.05). Within group, L:M increased marginally from 0.032 ± 0.006 at baseline, to 0.037 ± 0.010 pre-race and 0.054 ± 0.007 six days post-race with LAB^4^_ANTI_ (*p* > 0.05) Similarly, there was a non-significant increase in L:M with LAB^4^ from 0.028 ± 0.005 at baseline, to 0.039 ± 0.007 and 0.044 ± 0.012 both pre- and six days post-race respectively (*p* > 0.05).

However, for PL, L:M significantly increased over the intervention (F = 8.16, *p* = 0.004, ηp^2^ = 0.51) from 0.012 ± 0.008 at baseline to 0.041 ± 0.010 pre-race (*p* ≤ 0.05). L:M further increased in PL to 0.061 ± 0.011 six days post-race (*p* = 0.002). A similar interaction effect for time was also observed with the per cent recovery of lactulose (F = 5.66, *p* = 0.005, ηp^2^ = 0.14), as shown in Table 4. Within group, for PL only, the per cent recovery of lactulose increased from 0.35% ± 0.18% at baseline to 0.94% ± 0.12% six days post-race (*p* = 0.01). No significant differences were found for per cent recovery of mannitol either between or within groups (*p* > 0.05).

### 3.5. GI Questionnaire

Overall symptom counts for training related GI issues were significantly lower in both LAB^4^ groups at the end of Month 1 (7.80 ± 2.20 for LAB^4^_ANTI_ and 6.78 ± 1.31 for LAB^4^) compared with PL (11.90 ± 2.02; *p* ≤ 0.013). However, by Month 2, only symptom counts for LAB^4^ were significantly lower (8.11 ± 2.18) than PL (13.20 ± 2.72; *p* < 0.001). At Month 2, there was a significant increase in symptom counts for LAB^4^_ANTI_ (10.70 ± 2.88, *p* = 0.015 within group), which was also greater than LAB^4^ (*p* = 0.036). By the end of the intervention, there was a similar pattern to Month 1, with both LAB^4^ groups reporting lower symptom counts to training GI issues (8.80 ± 2.70 for LAB^4^_ANTI_ and 7.00 ± 2.16 for LAB^4^) compared with PL (13.90 ± 2.42; *p* < 0.001).

Average symptom severity was significantly lower with both LAB^4^ groups at Month 1 (9.80 ± 3.05 for LAB^4^_ANTI_ and 7.56 ± 1.56 for LAB^4^) compared to PL (15.50 ± 2.97; *p* < 0.001). This pattern continued throughout the intervention, with severity scores for LAB^4^ groups remaining lower than PL (16.70 ± 3.64) at Month 3 (10.10 ± 3.27 for LAB^4^_ANTI_ and 8.00 ± 2.50 for LAB^4^; *p* < 0.001). No differences were reported for average symptom severity within group across the intervention (*p* > 0.05) or between LAB^4^ groups (*p* > 0.05).

### 3.6. Race Times

**Figure 3:**
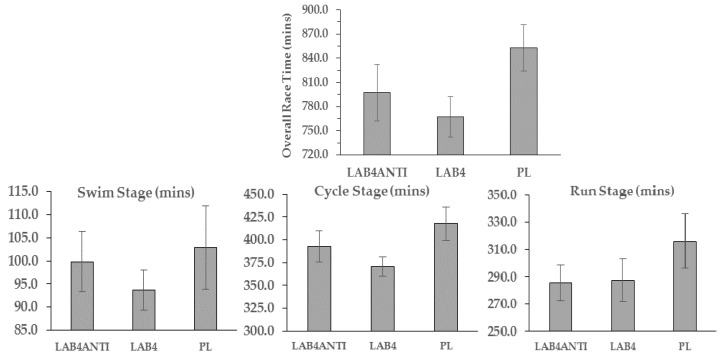
Race time comparisons in minutes (Mean ± SE), including triathlon stage disciplines. No significant differences reported between groups (*p* > 0.05). Converted overall times: LAB^4^_ANTI_ = 13 h 17 min 07 s (±34 min 48 s); LAB^4^ = 12 h 47 min 47 s (±25 min 31 s); PL= 14 h 01 min 40 s (±31 min 32 s).

Mean race finishing times are shown in Figure 3. Overall, no significant differences were found between groups for overall finishing times (F = 2.12, *p* = 0.149), despite faster completion times for both LAB^4^_ANTI_ (13 h 17 min 07 s ± 34 min 48 s) and LAB^4^ (12 h 47 min 13 s ± 25 min 06 s) compared with PL (14 h 12 min 51 s ± 29 min 54 s). Faster swim and cycle stage times were also recorded on average for LAB^4^ (93.7 ± 4.4 min and 370.7 ± 10.4 min respectively) compared with both LAB^4^_ANTI_ (99.8 ± 6.5 min and 392.5 ± 16.9 min) and PL (103.6 ± 9.9 min and 405.1 ± 14.3 min). However, average stage times were not significantly different between groups for either swim (F = 0.45, *p* = 0.642) or cycle (F = 2.30, *p* = 0.129) stages.

This was further reflected in the marathon stage, despite both LAB^4^ groups completing the marathon course in similar times (285.8 ± 13.1 min for LAB^4^_ANTI_ vs. 287.41 ± 16.04 min for LAB^4^) in contrast to PL (320.8 ± 21.1 min; F = 1.06, *p* = 0.368). It was however noted that despite a non-significant interaction effect, post-hoc comparisons indicated a significant difference for the bike stage between LAB^4^ and PL groups only (*p* = 0.046), and a strong trend for a significant differences in overall finishing times between these two groups (*p* = 0.058).

## 4. Discussion

The concept of exercise-mediated endotoxemia remains contentious, with varying terminology reported in the literature, including methodologies used to assess endotoxin units. Whereas some authors have referred to the term “mild” endotoxemia to reflect relatively small changes in endotoxin levels (5–15 pg·mL^−1^) along with acute cytokinemia following sustained endurance exercise [9], others have suggested that values ranging from 10 to 50 pg·mL^−1^ are indicative of normal, yet sustained “low grade” endotoxin levels which may modulate a systemic inflammatory response [13,16]. Clinical states of endotoxemia reflect much higher endotoxin concentrations (>80–300 pg·mL^−1^ [11,12]), with only a handful of studies demonstrating that strenuous ultra-endurance exercise actually elicits these levels at the point of exhaustion or during acute recovery [10,15]. Less is known whether repetitive GI provocation from repeated training elevates resting endotoxin levels, and what impact this may have on individuals preparing for, or recovering from, long distance events.

Average resting endotoxin units in the current study remained within normal limits (<10 pg·mL^−1^), and were comparable to values observed (~11.0 ± 5.0 pg·mL^−1^) for healthy volunteers with similar fitness levels [14,39,40]. This is in contrast to our hypothesis that plasma endotoxin units would be raised following repetitive endurance exercise as evidenced elsewhere [13,15]. However, the range for endotoxin units was 3.03–27.75 pg·mL^−1^, indicating that if “low grade endotoxemia” does occur at values >10–50 pg·mL^−1^, then some individuals may be susceptible to repeated exposure.

LPS translocation across the GI tract is known to provoke systemic immune reactions with varied consequences [41]. Specifically, LPS attachment to LPS-binding protein and its transference to an MD-2/toll-like receptor (TLR) 4/CD14 complex activates NF-kappa-B and various inflammatory modulators (TNF-α, IL-1β, IL-6 and CRP). This is considered a protective mechanism acting to minimise bacterial entry across the GI tract. Under normal physiological conditions, endotoxins from gram negative bacteria are usually contained locally, with only relatively small quantities entering the systemic circulation. However, when GI defences are either disrupted (i.e., luminal damage from exercise) or LPS “sensing” is “overloaded” a heightened inflammatory response may result which could, in part, relate to GI symptoms associated with exercise [42]. This could have implications to daily recovery mechanisms throughout prolonged training periods, and in the days following ultra-endurance events.

The use of a 12-week LAB^4^ strategy reduced average endotoxin units by 26.0%, but was not statistically significant. In contrast, the LAB^4^_ANTI_ intervention resulted in a significant 46.6% reduction in endotoxin units, with pre-race levels in this group reducing to 4.37± 0.51 pg·mL^−1^. These levels are comparable to resting values observed in trained athletes elsewhere at ~3.8 ± 2.0 pg·mL^−1^ [13], and could have important implications for those individuals with previously raised endotoxin levels (e.g., >20 pg·mL^−1^) or who are more susceptible to training related GI symptoms. Whilst the general trend in IgG anti-EU supported these findings, the inter-individual variability observed resulted in non-significant findings. It was noted that average IgG anti-EU levels for LAB^4^_ANTI_ were, however, significantly lower than both LAB^4^ and PL. Although this possibly indicates an adaptive response in this group, IgG anti-EU ranges observed were comparable to those reported elsewhere [43] and most likely reflect variance in relation to individual gut microbiota profiles.

Altered GI permeability was only observed in the PL condition, which whilst not reaching clinical significance (i.e., L:M ≥ 0.09; [44,45]), represented a 4.2 fold increase over the intervention and recovery periods (compared to a 0.7 fold increase in the L:M ratio for LAB^4^_ANTI_ and 0.6 fold increase for LAB^4^). Additionally, both GI symptom count and severity were significantly lower in both LAB^4^ interventions compared with PL by the end of the training period, observations similar to those reported elsewhere employing probiotic strategies [21,46,47]. Collectively, these results support the contention that a multistrain pro/prebiotic intervention maintains tight junction stability, potentially through interference with phosphorylation processes. Although this supports previous findings [26,48], such strategies may only apply to chronic interventions, as recent research has demonstrated no impact of acute (7 days) probiotic use on endotoxin levels following endurance exercise at 60%VO_2_max under ambient or heat-stressed conditions [49].

Studies have demonstrated that regular use of probiotics can improve epithelial resistance by establishing competitive “biofilm” activity. Indeed, as LPS types vary across gram-negative bacteria species, some LPS are poorly sensed by TLR4 and may have more direct impact on NF-κ-B activation [50]. Therefore, direct exclusion of LPS translocation through maintained epithelial integrity and/or increased preponderance of gram-positive genera may offer potential therapeutic benefit [51]. Specifically, the provision of *Lactobacillus* genus may work by activating TLR2 and hence more favourable innate immune responses [52,53,54]. Additionally the use of a 14 week multistrain probiotic strategy significantly decreased faecal zonulin levels elsewhere, supporting improved tight junction stability [26].

However, effects of probiotics are strain specific. The product used in the current study does not appear to have been used in a training context previously. Clinical trials, however, have demonstrated that the inclusion of the *Lactobacillus* strains CUL-60 and CUL-21 modulated the facultative anaerobes (*Enterobacteriaceae*, *Enteroccus/Streptococcus* and *Staphylococcus* species) during antibiotic therapy [24,55]. Two other papers utilizing similar dosages to the current study also indicate that the CUL-60 and CUL-21 strains prevented an increase in antibiotic resistant *Enterococci* and reduced the incidence of *Clostridium difficle* toxins [25,56]. Future research should address strain specific colonization and impact on gut microbiota, which may explain inter-individual differences particularly in athletes.

The inclusion of *Bifidobacterium* and prebiotics (e.g., inulin, galacto- or fructo-oligosaccharides [FOS]) in such formulas may also provide additional benefits. Studies have demonstrated a significant increase in short-chain fatty acids (SCFA), with prebiotic use additionally supporting increased *Bifidobacteria* growth, and decreased levels of bacteriodes and *Fermicutes* phyla [57,58,59,60,61]. Additionally, prebiotic use has been shown to improve mucosal dendritic cell function associated with TLR2 activity [62], and increase the expression of glucagon-like peptide 2, associated with GI barrier regulation [59]. Although low dose FOS was employed in the current study, the “synbiotic” effect with a multistrain probiotic formula has been shown to confer improvements in gastrointestinal well-being elsewhere [63].

In the current study, a combined antioxidant in conjunction with a multistrain pro/prebiotic strategy appeared to confer an additive effect through reduced endotoxin unit levels at the end of the 12-week training period, as well as six days post-race. Specifically, alpha lipoic acid acts as a multi-functional antioxidant through rapid regeneration of glutathione [64,65]. GI epithelial damage may be directly associated with oxidative stress from GI ischemia (particularly hydrogen peroxide), and in extreme cases may lead to ischemic colitis or infarct tissue [66]. Endogenous glutathione peroxidase may be a crucial enzyme in the protection of the intestinal lumen from repetitive damage [67]. Alpha lipoic acid, along with *N*-acetyl-carnitine, may therefore act in a local antioxidant manner, and via phosphoinositide 3-kinase/Akt signalling may down-regulate LPS stimulation of NF-κ-B [68,69,70]. Other dietary antioxidants such as ascorbic acid have been shown to blunt the endotoxin response to exercise, but with secondary effects on ascorbate radical production [28]. Various flavonoids (e.g., quercetin found in onions) and isoflavones (e.g., genistein found in soybeans) have been shown to inhibit protein kinase C and protein tyrosine kinases respectively, although the use of 2 g·day^−1^ quercetin for seven days was also shown to block the rise in heat shock protein 70, potentially restricting thermotolerant adaptation [71].

To date, only two studies appear to have assessed endotoxin levels in the hours/days following ultra-endurance events. Subclinical symptoms associated with exercise-mediated endotoxemia may vary in both severity and duration (possibly lasting several days). One study demonstrated raised (but effectively normal) endotoxin levels at 16 h following an ironman triathlon, but did not assess return to baseline levels [9]. A further study demonstrated that endotoxin levels had returned to baseline 1–3 weeks post event, reflecting the exhaustive nature of the event [10]. A limitation of the current study was the logistical difficulty of collecting samples immediately or 24 h post event. With varying individual travel plans, participants were instructed to rest in the 5 days post-race. At six days post-race, endotoxin units remained unchanged with PL (8.02 ± 1.14 pg·mL^−1^), but were significantly lower for both intervention groups (5.18 ± 0.57 pg·mL^−1^ for LAB^4^_ANTI_, and 5.01 ± 0.28 pg·mL^−1^ for LAB^4^). This represented an overall reduction in endotoxin units from baseline of 36.8% for LAB^4^_ANTI_ and 43.9% for LAB^4^ strategies. Although cytokine profiles were not assessed in the current study, a general reduction in endotoxin levels via pro/prebiotic/antioxidant combinations may have important benefits in minimizing low grade cytokinemia from endurance exercise [72,73].

The use of LAB^4^_ANTI_ or LAB^4^ did not significantly improve times within-race only in direct comparison to PL. This is despite a 6.5% (~56 min) faster overall time for LAB^4^_ANTI_ compared to PL, and 10.0% (~86 min) faster for LAB^4^ compared to PL. This did not reflect our original hypothesis, and likely reflects the wider variance in capabilities observed with “recreationally trained” individuals. However, it was noted that faster times were reported for LAB^4^ during both swim and cycle stages, with a trend towards an overall difference compared to PL (*p* = 0.058). It is acknowledged that exercise performance was not assessed in the current study as baseline measures could not be ascertained. As participants were entering their first long distance triathlon, comparison between groups only provided an insight into whether either intervention strategy offered potential race benefits. Future research should focus on whether combined pro/prebiotic/antioxidant strategies offer direct performance benefits in controlled settings, particularly in individuals more susceptible to GI related issues.

## 5. Conclusions

Chronic multistrain pro/prebiotic supplementation during periods of endurance training may provide individual support to minimise GI symptoms through maintenance of intestinal permeability. The inclusion of an antioxidant strategy (e.g., α-lipoic acid/*N*-acetyl carnitine) may confer additive benefits via reductions in training-related endotoxin unit levels. In a recreationally trained cohort, LAB^4^_ANTI_ or LAB^4^ strategies did not influence race times in direct comparison to a control group also undertaking their first long distance triathlon. Combined pro/prebiotic/antioxidant strategies may have important implications for individuals undertaking endurance training, particularly those more susceptible to GI symptoms.

## Figures and Tables

**Table 1:** Pre-screening (Month 0) and baseline (Month 6) characteristics for intervention groups.

Variable	LAB^4^_ANTI_		LAB^4^		PL	
Distribution	(*n* = 10; 7 male, 3 female)		(*n* = 10; 9 male, 1 female)		(*n* = 10; 9 male, 1 female)	
Age (years)	33 ± 2		35 ± 2		35 ± 3	
Height (m)	1.74 ± 0.34		1.79 ± 0.27		1.76 ± 0.16	
	Pre-screening	Baseline	Pre-screening	Baseline	Pre-screening	Baseline
Weight (kg)	75.21 ± 4.12	73.61 ± 3.96 *	83.77 ± 4.71	81.94 ± 4.44 *	77.42 ± 3.03	74.56 ± 2.76
Body fat (%)	22.56 ± 1.67	19.36 ± 2.23 *	21.88 ± 1.68	20.93 ± 1.52	21.28 ± 2.38	18.64 ± 1.93 *
VO_2_max (L·min^−1^)	3.26 ± 0.20	3.57 ± 0.19 *	3.78 ± 0.28	3.94 ± 0.27	3.30 ± 0.14	3.70 ± 0.10 *
VO_2_max (mL·kg^−1^·min^−1^)	42.90 ± 1.59	48.60 ± 1.80 *	43.89 ± 1.75	47.56 ± 1.69 *	43.40 ± 2.53	50.50 ± 1.71 *

Data presented as mean ± SE No significant differences reported between groups. * denotes significant difference (*p* < 0.05) to pre-screening only within group.

**Table 2:** Dietary analysis comparisons between groups.

Variable	LAB^4^_ANTI_	LAB^4^	PL
Energy intake (kcal·kg^−1^·day^−1^)			
T1	35.96 ± 2.16	33.13 ± 1.16	35.57 ± 2.88
T2	33.88 ± 2.06	33.03 ± 4.66	34.57 ± 2.98
T3	35.42 ± 2.57	35.76 ± 2.43	36.60 ± 2.85
Fat (g·kg^−1^·day^−1^)			
T1	1.45 ± 0.13	1.28 ± 0.10	1.24 ± 0.14
T2	1.37 ± 0.12	1.27 ± 0.21	1.19 ± 0.08
T3	1.52 ± 0.18	1.26 ± 0.12	1.47 ± 0.11
Carbohydrate (g·kg^−1^·day^−1^)			
T1	4.29 ± 0.33	3.80 ± 0.30	4.46 ± 0.47
T2	3.95 ± 0.43	3.90 ± 0.59	4.32 ± 0.58
T3	3.93 ± 0.39	4.36 ± 0.36	4.34 ± 0.57
Protein (g·kg^−1^·day^−1^)			
T1	1.51 ± 0.12	1.43 ± 0.14	1.81 ± 0.18
T2	1.56 ± 0.07	1.39 ± 0.17	1.70 ± 0.14
T3	1.57 ± 0.15	1.68 ± 0.28	1.67 ± 0.19

Data represent average daily intake (mean ± SE). T1–3 represent Months 1–3 respectively. No significant differences reported between or within groups (*p* > 0.05).

**Table 3:** Training load comparisons.

Variable	Target Range	LAB^4^_ANTI_	LAB^4^	PL
*Weekly training load (AU)*				
T1	2173–2716	2410 ± 242	2851 ± 279	2807 ± 368
T2	2622–3278	3885 ± 558 #	4311 ± 348 *#	3915 ± 516 #
T3	2231–2789	2232 ± 148	2768 ± 498	2263 ± 180
*Training monotony (AU)*				
T1	1.07–1.33	0.94 ± 0.11	0.98 ± 0.08	1.11 ± 0.08
T2	0.97–1.21	0.88 ± 0.07	0.96 ± 0.08	0.88 ± 0.08
T3	1.27–1.58	0.90 ± 0.12	0.87 ± 0.09	0.72 ± 0.05
*Training strain (AU)*				
T1	2951–3688	2755 ± 562	2945 ± 450	3224 ± 566
T2	3350–4187	3430 ± 620	4065 ± 381 *#	3293 ± 552
T3	3352–4440	2281 ± 370	2681 ± 650	1946 ± 186

Data represent arbitrary units (AU) and presented as mean ± SE Target range indicates 80%–100% of training programme. No significant differences reported between groups (*p* > 0.05). * denotes significant difference from T1 within group (*p* ≤ 0.006). # denotes significant difference from T3 within group (*p* ≤ 0.04).

**Table 4:** Recovery of urinary lactulose and mannitol (%).

Variable	LAB^4^_ANTI_	LAB^4^	PL
*% recovery of lactulose*			
Baseline	0.71 ± 0.13	0.52 ± 0.07	0.35 ± 0.18
Pre-race	0.55 ± 0.14	0.74 ± 0.10	0.72 ± 0.17
6 days Post-race	0.83 ± 0.11	0.90 ± 0.24	0.94 ± 0.12 *
*% recovery of mannitol*			
Baseline	29.99 ± 2.87	25.57 ± 2.22	30.61 ± 3.96
Pre-race	23.31 ± 3.60	27.42 ± 3.33	23.51 ± 2.18
6 days Post-race	22.42 ± 3.27	25.01 ± 1.89	23.48 ± 2.64

Data presented as mean ± SE. * denotes significant difference to baseline within group only (*p* = 0.01).
